# The link between microplastics and colorectal cancer

**DOI:** 10.1007/s10555-026-10361-y

**Published:** 2026-07-31

**Authors:** Anisha Lynch-Godrei, Christopher Leung

**Affiliations:** 1 Department of Medicine, The University of Melbourne, Parkville, VIC 3010 Australia; 2 Walter and Eliza Hall Institute of Medical Research, Parkville, VIC 3050 Australia; 3 Monash Health, Clayton, VIC 3168 Australia; 4 Department of Gastroenterology and Hepatology, Austin Health, Heidelberg, VIC 3084 Australia; 5 Doctors for the Environment Australia, Carlton, VIC 3053 Australia

**Keywords:** Colorectal cancer, Microplastics, Nanoplastics, Literature review

## Abstract

Microplastics and nanoplastics (MNPLs) are known to enter the human body and accumulate within different tissues. As MNPLs have been shown to exert toxic effects *in vivo*, we aimed to evaluate the link between MNPLs and colorectal cancer tumorigenesis. A literature review was conducted, using Ovid MEDLINE and PubMed databases. MeSH terms “colorectal carcinoma” and “microplastic” were the foundations for the search strategy. Forty-one studies were initially identified, of which fourteen were ultimately selected for analysis. MNPLs are found within human colorectal tumors and are present at higher levels in the feces of colorectal cancer patients. The tissue sample studies however are limited by small sample sizes (*n = *10 or 25), and are representative of mainly Chinese populations (five of the six human studies conducted in China). The mouse studies observed increased tumor loads and dysbiosis in mice exposed to MNPLs, and the *in vitro* studies observed increased reactive oxygen species (ROS) when cells were exposed to MNPLs in culture. Although the direct link between MNPLs and *de novo* development of colorectal cancer remains to be elucidated, these findings suggest that MNPLs can induce the molecular changes involved in tumorigenesis, and tumorigenesis itself when combined with a compromised gastrointestinal barrier. Given that barrier disruption occurs with inflammatory bowel disease, smoking, alcohol, stress, and infections, these populations may be most vulnerable to the effects of MNPLs.

## Introduction

In the early twentieth century, the advent of plastic polymers was instrumental to driving forward the next industrial revolution. Over a hundred years later, we are now beginning to recognize the impact of these “forever-materials”, as they accumulate in landfills, soil, and waterways. While the plastics themselves pose an environmental hazard, their ability to break down and create microplastics and nanoplastics has become another source of concern in recent years. Microplastics and nanoplastics (MNPLs) are small particles of plastics that come about either from the intentional production of microplastics for use in hygiene products or medicine (termed primary microplastics), or through the degradation of whole plastic products via ultraviolet radiation, abrasive shearing, or chemical breakdown (termed secondary microplastics) [1]. These particles are further categorized based on their size, where microplastics include particles from 1–5000 μm, and nanoplastics are particles from 1–1000 nm [2].

Though plastics are ubiquitous in our daily lives, they may enter the body via inhalation, dermal contact, and through the digestive tract [1]. Direct consumption of MNPLs has the potential for the greatest impact on the body given the many sources of MNPL contamination in our diets. This connection seems obvious when we think about seafood, as we have known for a while about the significant plastic (and thus MNPL) pollution of oceans and waterways which ultimately end up in fish and crustaceans of human diets. However, more recent evidence suggests that we also obtain microplastic contamination from food packaging [3, 4], and perhaps more surprising is the contamination we get directly from fruits and vegetables grown in MNPL contaminated soil [5, 6].

Having gained a clearer picture of the sources of MNPL contamination encountered in daily life, along with the finding that we excrete various types of microplastics in our feces [7], has raised concerns over the potential impact that MNPLs may have on human health. Research involving animal models has suggested that MNPLs exert toxic effects. Studies on zebrafish exposed to polystyrene/polyethylene MNPLs in their aquatic environment have shown that plastic particles do accumulate within tissues, and that accumulation was associated with increased reactive oxygen species (ROS) production [8, 9]. Given that ROS is a known mediator of inflammation and DNA damage, it’s likely that MNPLs have the capability to influence tumorigenesis. Similar findings have also been reported in mammalian models, as accelerated tumor growth was observed in a mouse model of ovarian cancer after the addition of polystyrene nanoplastics to their drinking water [10].

Considering ingested MNPLs are exposed to harsh digestive conditions and directly contact the gastrointestinal epithelium, it's logical that the gastrointestinal system may be most vulnerable to the effects of MNPLs. Recent reports describe an alarming increase in the incidence of early-onset colorectal cancer (affecting people under 50 years old) over the 2013–2017 period [11]. This trend was strongest in Western countries like Australia, the Unites States of America, and New Zealand, where rates of late-onset colorectal cancer (affecting people over 50 years old) are instead declining [11]. Work by the Research in Early Age Colorectal Cancer Trends (REACCT) Collaborative also find that early-onset CRC cases are sporadic with genetic predisposition having little influence, which points to environmental factors being the likely cause for the increased incidence. They identify factors like modern Western diets, overuse of antibiotics, obesity, and changes in the gut flora as possible risk factors [12]. As we now recognize the significant amount of microplastics that pass through the digestive tract we wondered whether these particles had any impact on cancer development. Here we aim to evaluate the research describing the relationship between gut exposure to MNPLS and colorectal cancer tumorigenesis.

## Methods

The online databases Ovid MEDLINE and PubMed were selected for their relevance to our research question. Searches were conducted during October 2025, and included the MeSH terms “colorectal neoplasm”, and “microplastic”. Appendix Table 2 outlines the exact search strategy and Boolean operators used for the searches on each database. Article titles were scanned, duplicates omitted, and only articles with available full texts were further analyzed (reviews, conference proceedings, books, encyclopedia entries, letters to the editor, or editorials were omitted). Abstracts were then assessed for potential relevance before full texts were analyzed.

Inclusion criteria consisted of 1) articles written in English, 2) articles published within the last 20 years, 3) articles that evaluated the relationship between micro- and/or nano-plastics and colorectal tumorigenesis. Exclusion criteria included any articles that did not assess tumor development, or known tumorigenesis pathways in colonocytes, in relation to exposure of MNPLs.

## Results

**Fig. 1:**
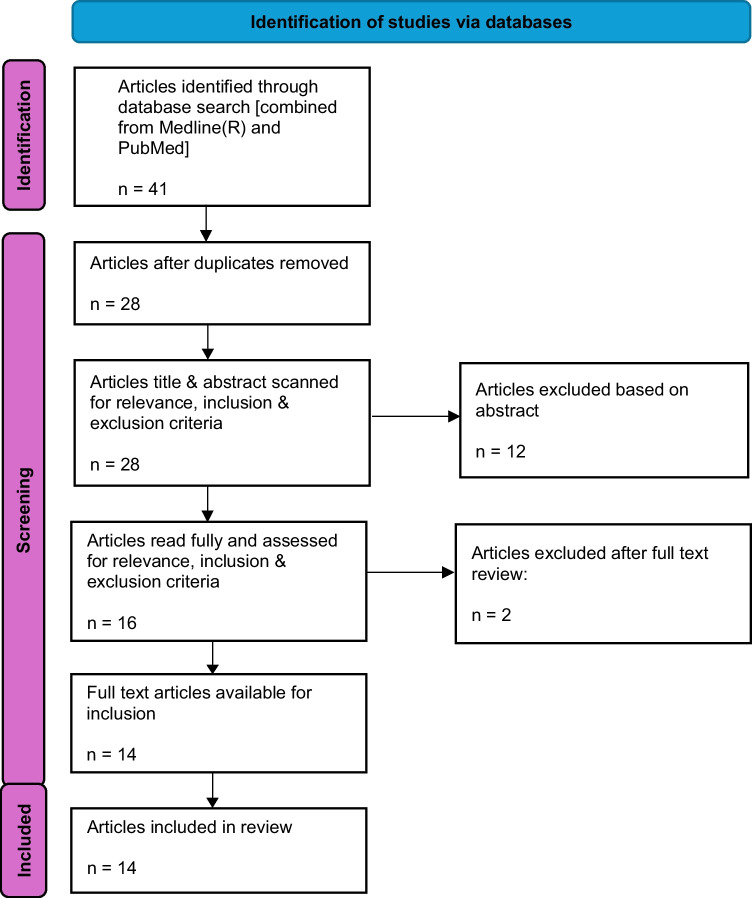
PRISMA flow diagram outlining the selection process for the articles in this study

Our search strategy yielded 41 articles from both Ovid MEDLINE and PubMed databases. After removal of duplicates there were 28 articles remaining which moved on to the abstract scanning phase. Titles and abstracts were then assessed according to our inclusion and exclusion criteria. This left 16 articles to undergo full-text analysis, for which two articles were excluded in the process. In all, fourteen articles met the inclusion criteria, were available as full texts, and were ultimately selected for comprehensive evaluation in this study. Summary outlined in Fig. 1.

From the fourteen articles included in this study, five included cross-sectional human studies on CRC tissue samples, one was a cross-sectional human study of the feces from CRC patients and controls, five of them involved *in vivo* experiments on mice (including wildtype mice, mouse models of colitis, and mouse models of colorectal cancer), and ten studies included *in vitro* experiments involving cells in culture. The cell lines included for these experiments include: six studies on Caco-2 cells, three on HCT116 cells, two on LoVo cells, two on HT29 cells, one on RAW264.7 macrophage cells, one used N9 microglia cells, and one used SW480 cells. Caco-2, HT29, HCT116, LoVo, and SW480 are all of colorectal cancer origin. A summary of the study design, MNPLs used, and the relevant findings can be found in Table 1.

**Table 1:** Summary of MNPL particle type/size and impact on tumorigenesis

Reference	Particle type	Particle size	Experimental model	Findings
[13]	PE, PP, PA, PC in both populations. PET in only Malaysian, and ABS in only USA samples	< 5000 μm	**Humans:** Colorectal cancer (CRC) samples from patients in Seattle, Washinton, USA between 1993–9 (*n = *25), & samples from CRC patients in Kota Bharu Malaysia collected between 2023–4 (*n = *25) were assessed for MNPL quantification and characterization using micro-Fourier Transform Infrared Spectroscopy (FTIR) and scanning electron microscopy (SEM). Ages not reported	• All samples contained MNPLs • US samples had an average of 25.0 ± 40.6 particles, while Malaysian samples had 32.2 ± 48.1 particles • Fibres were the most abundant shape in both, though US samples were longer and more brittle • Malaysian samples had smaller MNPLs (0–1000μm) compared to US samples (1001–5000μm)
[14]	**Humans:** PVC & PA **Mice:** not reported **Cell lines:** not reported	**Humans:** < 100 μm **Mice:** not reported **Cell lines:** not reported	**Humans:** Quantification and characterization of MNPLs in colorectal tumor tissue using Laser Direct Infrared (LDIR) chemical imaging spectroscopy, and SEM from 10 patients in Tianjin, China. Ages not reported **Mice:** MNPLs given by oral gavage to BALB/c-nu mice inoculated with LoVo colorectal cancer cells. Subsets of mice also received oxaliplatin **Cell lines:** HCT116 and LoVo colorectal cancer cell lines treated with MNPLs. Experiments also repeated after clatherin siRNA knockdown	• All tumor tissue contained MNPLs—predominantly PVC and PA, and particle sizes were mostly 0–100 μm • Both cell lines showed NF-kB mediated ↑ fatty acid uptake and oxidation after MNPL exposure (associated with ↑ CD36 & CPT1A) • Cell lines treated with MNPLs exhibited ↓ pyroptosis, thus resistance to cell death • Clatherin knockdown in cells led to ↓ MNPL uptake • Ingestion of MNPLs by mice resulted in ↑ tumors mass, which was resistant to oxaliplatin treatment, & associated with ↑ NF-kB activity and ↓ pyroptosis
[15]	PS	4 μm	**Mice:** BALB/c mice exposed to low or high doses of MNPLs in drinking water were evaluated for changes in gut microbiota. Mouse fecal samples also exposed to MNPLs in culture (as only source of carbon for energy) to assess for MNPL breakdown by SEM	• MNPL exposure led to shortening of the gastrointestinal tracts • Both high and low dose MNPL treated groups had ↑ in *Lepagella* and *Desulfovibrio* species • Positive association seen between ↑ *Desulfovibrio* & ↑ biotin production and ↑ sulphur-relay pathways (metabolite linked to cancer) • After exposure to fecal microbiota MNPL particles displayed slight degradation
[16]	PET, PA, PE, PC, PP	< 200 μm	**Humans:** Evaluation of MNPL levels in the feces of 258 CRC patients (aged 57.2 ± 12.2) and 493 healthy controls (aged 61.3 ± 10.7) in Quzhou, China using Agilent 8700 LDIR chemical imaging spectroscopy	• ↑ MNPLs found in CRC patient stool—43 particles/g dry weight in controls vs. 62 in CRC • Multivariable regression analysis showed a dose–response association between ↑ MNPLs and risk of CRC, and ↑ risk of CRC in females, people who eat spicy food, and people who eat fast food > 2 times a week
[17]	**Humans:** PVC **Cell lines**: PS	**Humans:** 0–30 μm **Cell lines**: 80 nm	**Humans:** Quantification and characterization of MNPLs in colorectal tumor and adjacent non-tumor tissue from 10 patients in Tianjin (aged 69.9 ± 9.07), China using Agilent 8700 LDIR chemical imaging spectroscopy, and SEM **Cell lines:** Knock-down of clatherin in MNPL treated HCT116 and LoVo colon cancer cell lines	• Non-tumor and tumor tissue both contained MNPLs (3/5 patients had ↑ MNPLs in non-tumor tissue vs tumor tissue) • Tumor tissue tended to have smaller MNPLs (0–30 μm) & were predominantly PVC • ↑ CA19-9 and CEA levels correlate with ↑ MNPLs • In vitro studies showed ↓ clatherin levels led to ↓ levels of internalized MNPL
[18]	**Humans:** PP, PE, PA, & PET **Mice:** Not reported **Cell lines:** Not reported	**Humans:** < 200 μm **Mice:** Not reported **Cell lines:** 60–80 nm	**Humans:** Quantification and characterization of MNPLs in colorectal tumor from 10 patients in Tianjin, China using Agilent 8700 LDIR chemical imaging spectroscopy, and SEM (no ages reported) **Mice:** MNPLs given by oral gavage to: **1)** female BALB/nu mice subcutaneously injected with HCT116 cells, and **2)** female C57/bl6 mice injected with azoxymethane and dextran sulphate sodium (AOS + DSS) **Cell lines:** HCT116 and SW480 colon cancer cell lines grown in media with MNPLs,	• All human samples contained MNPLs. Most abundant types in human CRC: PP, PE, PA, & PET • BALB/nu + HCT116 mice had ↑ tumor growth and oxaliplatin resistance • AOM + DSS mice exposed to MNPLs had more CRC tumors & microbiota dysbiosis (↑ in *Clostridiales* and *Lachnoclostridium*) • MNPLs on cell lines had ↑ proliferation & invasion (ie. metastasis), induction of protective autophagy (↑ mTOR, ULK1, LC3B, and SQSTM1), & resistance to oxaliplatin chemotherapeutic
[19]	PS	20 nm	**Mice:** BALB/c, AOS + DSS model of colitis, given low, medium, or high concentrations of nanoplastics by oral gavage for 16 weeks **Cell lines:** Lipopolysaccharide (LPS) treated Caco-2 cells (human adenocarcinoma)	• AOS + DSS mice receiving medium and high amounts of MNPLs had ↑ tumor loads & dysbiosis (↑ *Allobaculum*, and ↓ *Lactobacillus)* • Mice and cell lines exposed to MNPL had ↑ levels of reactive oxygen species (ROS), ↑ cell proliferation, ↑ DNA damage, and ↑ activation of the PI3K/AKT/mTOR pathway (reversible in cells with fenofibrate treatment) • MNPL exposure ↑ lipid peroxidation, ↓ fatty acid metabolism (↓CPT1A & ATGL)
[20]	Environmentally weathered plastics (PET, PP, HDPE, PVC, nylon)	Not reported	**Cell lines:** Caco-2 exposed to plastic-associated chemicals dissolved in DMSO (functional additives, byproducts, and metals)	• PVC DMSO extract ↑ toxicity, which increased with ↑ conc. • ↑ ROS with nylon exposure • Nylon, PVC, and HDPE DMSO extracts induced DNA damage • PVC—↑ metal and PAH contamination • HDPE and PET— ↑ PAH contamination
[21]	PS, PVC, PE	Not reported	**Humans:** MNPL content assessed in 61 tumor samples from patients in Guangzhou, China with the following cancers: lung (10), cervical (12), gastric (10), colorectal (10; aged 52–71), esophageal (9), and pancreatic (10). Analysis via pyrolysis–gas chromatography-mass spectrometry	• MNPLs identified in 26 of the 61 samples • Colorectal tumors had 5/10 MNPL positive samples. Gastric cancer had 4/10, & esophageal had 0/10 • MNPL type in CRC were PS, PVC, and/or PE
[22]	PS	25, 50, & 100 nm	**Cell lines:** HT29 (human colon adenocarcinoma) and N9 (mouse microglia) cells	• N9 cells, but not HT29 cells, have ↑ cell death in response to smallest nanoplastics • Nanoplastics promote chronic inflammatory state by inducing inflammatory cascades of immune cells (↑ iNOS expression) and by priming gut epithelia to become more sensitive to inflammation (↑ TLR4 receptors, and ↑ NFκB signalling)
[23]	PE, & PS	~ 500 nm	**Mice:** BALB/c mice—colons injected with CT26-Luc carcinoma cells, and given 10 mg/kg of PE by oral gavage for 7 days **Cell Lines:** RAW264.7 (macrophage) and Caco-2 cells treated with NPs	• In mice, NP exposure associated with gut shortening, gut barrier damage, and ↑ tumor growth. PE/PS ↑ inflammation (via macrophage IL-1β secretion) but also induced immunosuppression (↓ tumor infiltrating CD8^+^ & CD4^+^ cells, and ↑ Treg population) • NPs did not affect viability of Caco-2 or RAW264.7 cells • RAW264.7 cells had lysosomal dysfunction after NP phagocytosis, & ↑ ROS, IL-1β release
[24]	PE	5–60 μm	**Cell lines:** Human colon adenocarcinoma Caco-2 and HT-29 cells grown in the presence of **1)** MPs, and **2)** ethanol extracts from MPs	• Dose-dependent effect of PE on viability, cytotoxicity (LDH), and mitochondrial superoxide production in both cell lines • Extracts from PE particles ↑ mitochondrial superoxide in both cell types, but viability & cytotoxicity only impacted in Caco-2 cells
[25]	PS + PFOS	20 nm, 100 nm, and 1 μm	**Cell lines:** Caco-2 cells incubated with PFOS alone, as well as PFOS + PS particles	• PFOS facilitates internalization of MNPLs (30% ↑) • MNPLs reduced PFOS bioavailability, thus reducing their toxicity • 20 nm particles had ↑ cell entry and ROS production • 1 μm particles ↑ membrane damage, ↑ mitochondrial stress
[26]	PU	Not reported	**Cell lines:** Caco-2 cells incubated with different conc. of PU MNPLs after exposure to **1)** UV light in air, **2)** UV light in water Cells also incubated with **3)** UV exposed PU-leachate	• UV-aging of PU causes ↑ particle conc. over time. UV exposure in air leads to ↑ conc. relative to water • Aging in different mediums did not affect type of particles released • Longer UV exposure times ↑ cytotoxicity of Caco-2 cells (likely by ↑ conjugated carbonyl & ROS) • PU-leachate had reduced cytotoxicity & membrane damage with longer UV times

*PS – polystyrene, PA – Polyamide, PET – polyethylene terephthalate, PP – polypropylene, PVC – polyvinyl chloride, HDPE – high density polyethylene, PU – polyurethane, PAH – Polycyclic aromatic hydrocarbons, ABS—Acrylonitrile butadiene styrene, LDH – lactate dehydrogenase, PFOS—Perfluorooctane sulfonate, NPs – nanoplastics, ROS – reactive oxygen species

** Table only describes relevant results to the aim of this study

Of the studies that included human CRC tumor samples, MNPLs were detected in 100% of the patient samples from four studies [13, 14, 17, 18], and in 50% of the CRC patient samples from the other study [21]. All of these studies had a fairly low sample size, between 10–25 patients, and the majority of these patients came from China. Differences were observed between the predominant types of MNPLs detected within tumors by each study. The three studies from Tianjin had differing results with regards to the types of MNPLs; one indicated that all their patients had mostly polyvinylchloride (PVC) and polyamide (PA) MNPLs within tumors [14], another detected the presence of most MNPL types but only found a significant increase in PVC levels in tumor tissue compared to adjacent healthy tissue [17], and the last reported polypropylene (PP), polyethylene (PE), PA, and polyethylene terephthalate (PET) as the main MNPLs types [18]. Interestingly, the study comparing CRC samples from US patients in the 1990s to samples from Malaysian patients from the 2020s found the presence of PE, PP, PA, and polycarbonate (PC) in all samples, however PET was only present in Malaysian samples [13]. Also of note was that Pan, Hao, and colleagues (2025) reported a correlation between increased amounts of MNPLs in the colon with elevated levels of CEA and CA19-9 cancer biomarkers. Specifically, elevated CEA was associated with higher PP levels in non-tumor tissue, while elevated CA19-9 was associated with higher PVC levels within tumor tissue [17].

From the single large-scale human study assessing MNPLs in the feces of patients with CRC compared to healthy controls they observed that all samples contained MNPLs, and that CRC patients on average had more MNPLs per gram dry weight than controls (62 particles vs 43 particles). Healthy controls had 30% PET, 16% PA, 14% PE, 12% PP, and 10% PC, whereas CRC patient samples had 37% PET, 25% PA, 16% PE, 6.4% PC, and 5.5% PP [16]. Statistical analysis via restricted cubic splines further demonstrated that increased MNPL levels found within feces was associated with an increased risk of developing CRC. Other variables identified for increasing risk of CRC when exposed to MNPLs included female sex, regular consumption of spicy food, and consuming fast food > 2 times per week [16].

**Fig. 2:**
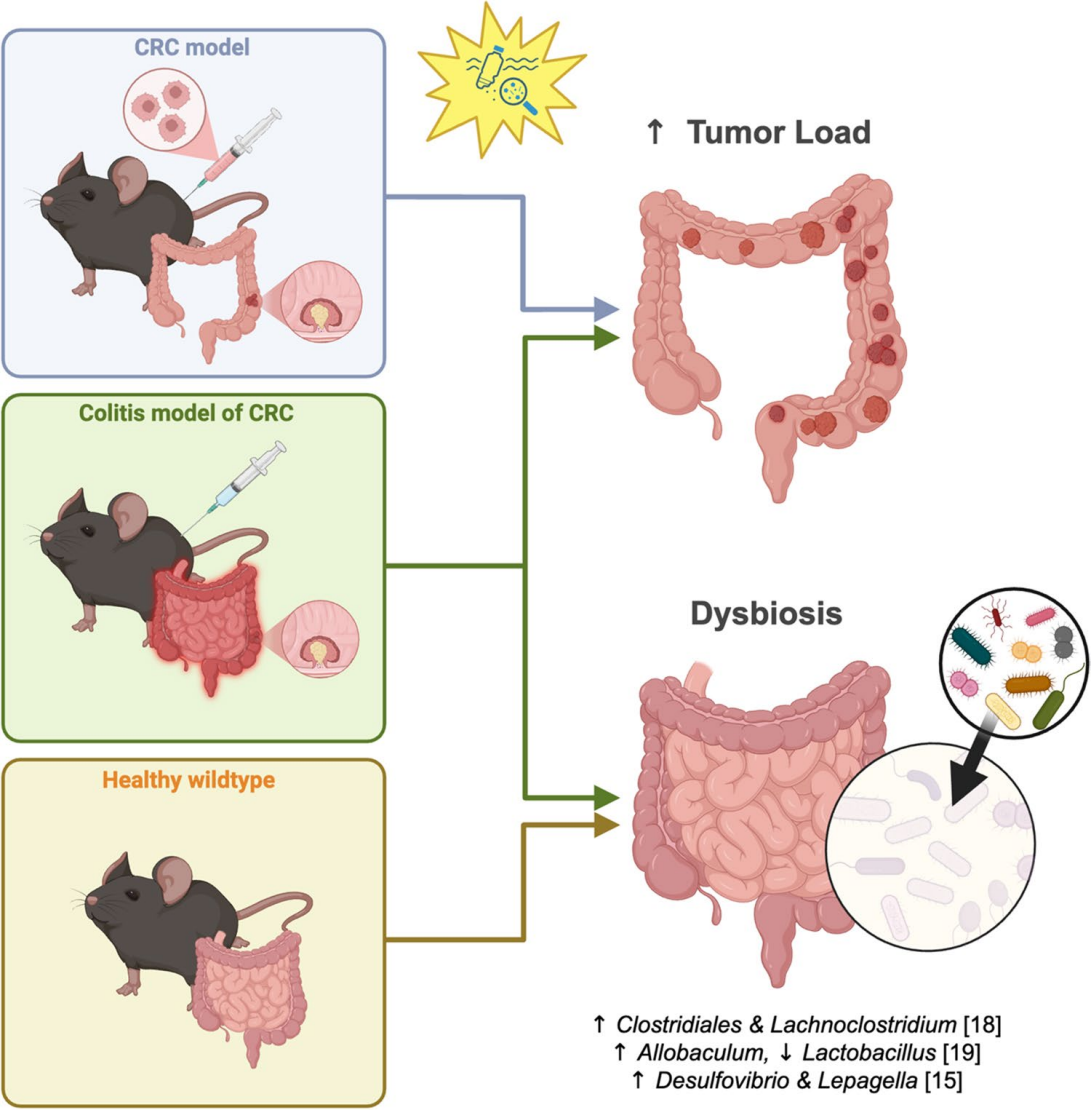
Microplastics and nanoplastics increase tumor load in mouse models of colorectal cancer and induce dysbiosis in colitis models of colorectal cancer and healthy wildtype mice [27]

All *in vivo* experiments evaluating CRC tumor growth observed increased tumor load in the mouse models exposed to MNPLs [14, 18, 19, 23]. The three studies that assessed the gut microbiome reported dysbiosis after exposure to MNPLs, however the changes to the gut flora they observed were different. Pan, Han and colleagues (2025) assessed the microbiome on the Azoxymethane and dextran sulphate sodium (AOM + DSS) model of colitis in C57/bl6 mice, and they saw that after administration of MNPLs by oral gavage there was an increase in gut bacteria of the *Clostridiales* and *Lachnoclostridium* species [18]. Tian and colleagues (2025) also used the AOM + DSS colitis model but on BALB/c mice [19]. They observed an increase in *Allobaculum* species and a decrease in *Lactobacillus* species after ingestion of 20 nm PS particles by oral gavage. Lastly, the only study to evaluate changes to the gut microbiome in healthy wildtype BALB/c mice after exposure to low or high doses of 4 μm PS particles in drinking water found an increase in *Lepagella* and *Desulfovibrio* species [15]. MNPL exposure was also associated with increased chemoresistance to oxaliplatin as reported by two *in vivo* studies [14, 18]. A summary of the *in vivo* findings can be seen in Fig. 2.

Across the ten studies utilizing cell lines to evaluate the effects of MNPLs, conflicting results were observed regarding cell viability. Two studies observed reductions in cell viability in the Caco-2 and HT29 CRC cell lines [24, 26], while another two studies reported no change in cell viability in those same two cell lines when exposed to MNPLs [22, 23]. Pan, Han, and colleagues (2025) reported increased HCT116 and SW480 cell proliferation in repones to MNPLs given at 25 µg/mL, which was associated with an increase in protective autophagy [18]. Conversely, a more recent study found differences in the growth and proliferation of HCT116 and LoVo cells based on the dose of MNPLs delivered [14]. Lower doses promoted proliferation (15 µg/mL) while higher doses were cytotoxic and led to reduced proliferation. They further go on to show that exposure to chronic low levels of MNPLs increased NF-kB-mediated fatty acid absorption and metabolism, which was associated with inhibited pyroptosis and thus increased survival of these cancer cells. These results were further confirmed in a mouse model of CRC [14].

Of the six studies that evaluated reactive oxygen species (ROS) production exposure, all found higher levels of ROS after MNPLs exposure (5 from colon cancer cell lines, and 1 from a macrophage cell line), with further DNA damage detected in Caco-2 cells from two studies that assessed DNA integrity [19, 20, 23–26]. Of note, Tian and colleagues (2025) also validated these *in vitro* findings of increased ROS and DNA damage in a mouse model of colitis [19]. Also of particular interest was that leachate extracted from either commercially available MNPLs or environmentally weathered plastics was sufficient to induce ROS production and DNA damage in Caco-2 cells [20, 24].

## Discussion

### The impact of MNPLs on colorectal cancer in humans

**Fig. 3:**
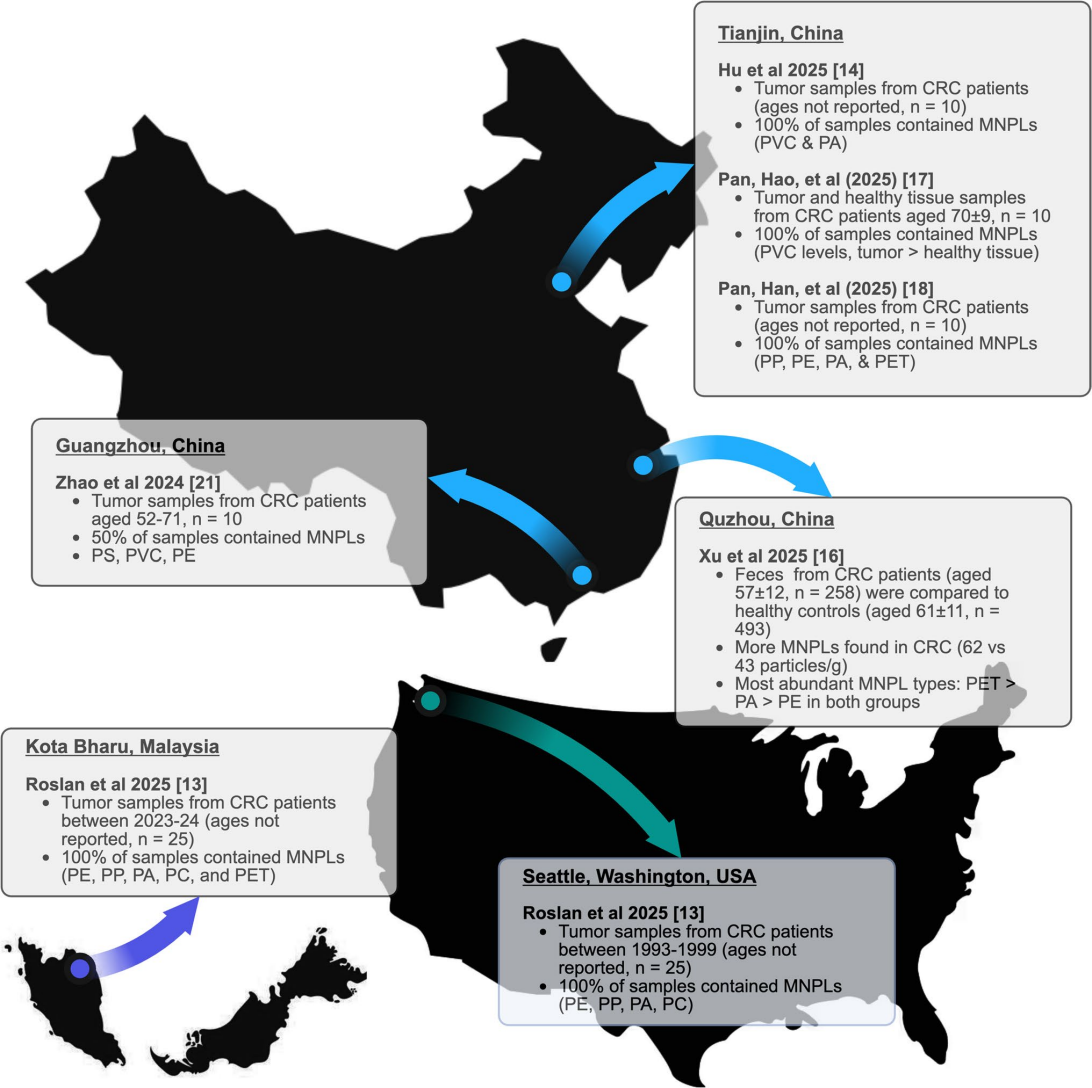
Summary of results from the cross-sectional human studies evaluated [30]

The human studies described here characterize the presence of MNPLs in 100% of CRC tumor tissues evaluated, apart from Zhao and colleagues (2024) which observed MNPLs in 50% (5/10) CRC samples evaluated [21], see Fig. 3 for a summary of the human studies. They go on to quantify and characterize the MNPLs present in CRC tissue, however only one of them made comparisons to adjacent healthy colon tissue [17]. Interestingly, there was no significant differences reported in the amount of MNPL particles in tumor and adjacent non-tumor tissue – and in fact in three of the five patient samples compared, there appeared to be more particles in the non-tumor tissue. These findings differ from that of a study conducted on colon adenocarcinoma samples collected from patients in Turkey – MNPLs were detected in 16 CRC patients (aged 50–70) and 15 healthy controls (aged 40–80), though they were found to be significantly higher within tumors [28]. Another study conducted on 11 patients undergoing colectomy in Malaysia also confirmed the presence of MNPLs in all specimens. Although two of these patients were not being treated for CRC—one had a bleeding arteriovenous malformation, and the other had a colon perforation [29]. This indicates that it is not abnormal for healthy colon tissue to contain a certain degree of MNPLs without causing CRC. This may instead suggest that other factors like MNPL size, MNPL type, or plastic additives (i.e. leachate compounds) may have a significant influence over CRC development. As we continue research in this area, we should however be wary of the potential of the tumor microenvironment to attract MNPLs. Which is to say, just because higher amounts of MNPLs are observed in CRC tumors does not mean that this represents a cause-and-effect relationship.

There were also differences reported between type of MNPLs found in tumor tissues between studies, which may be reflective of the different types of MNPLs people are exposed to by region and period of time. One study found significantly higher levels of PVC in CRC tumor tissue compared to adjacent healthy tissue, which might suggest that PVC has an increased risk of tumorigenesis in colonocytes [17]. However, this was only reported in a small sample size of 10 CRC patients from Tianjin, China. Furthermore, the data from the study evaluating MNPLs in feces from CRC patients and healthy controls only observed PVC as the 7th most abundant MNPL type in both groups [16]. While it is possible that we might expect to see differences in the types of MNPLs that get embedded in CRC tumors and the MNPLs that get excreted in feces, at this stage it’s difficult to draw any meaningful conclusions about specific MNPL types being more hazardous than others. Importantly, we should also bear in mind that these findings come from a small number of studies with very limited sample sizes (four studies with *n = *10 from participants only within China, and one study with *n = *25 from Malaysia and *n = *25 from USA).

As such we are not yet able to make generalizations about these findings until further research involving larger sample sizes and more diverse populations are conducted.

Perhaps the most impactful findings come from the first epidemiological evidence from a large-scale study to show that there are more MNPLs found within the feces of CRC patients compared to healthy controls [16]. Their statistical analysis also predicts that with increased MNPLs found in feces (thus increased consumption of MNPLs) comes a higher risk of CRC development. This is akin to 2022 study that found almost 50% higher levels of MNPLs in the feces of inflammatory bowel disease (IBD) patients compared to healthy controls in Nanjing, China [31]. Variables that may further amplify CRC risk include being of biologically female sex, as well as two modifiable factors including regular consumption of spicy food and eating take-out food twice or more per week. For now, these results indicate that people with CRC also have a higher environmental exposure to MNPLs through their diets. It would be important to build on this evidence in future, to assess MNPL levels within CRC patients’ tumor and healthy tissue in conjunction with the amount of MNPLs found in feces to help work out a possible cause-and-effect relationship.

With regards to the effect that MNPLs may have on early-onset CRC, the studies assessing human tissue evaluated here either do not report on the ages of CRC patients [13, 14, 18], or recruited only late-onset CRC patients [17, 21]. Thus at this stage we cannot draw any conclusions on the link between MNPLs and early-onset CRC. Future work should aim to compare MNPLs in early-onset CRC patients, late-onset CRC patients, and healthy controls to help elucidate whether higher MNPL burden, type, size, or shape of MNPL is linked to CRC tumorigenesis earlier in life. There is also emerging evidence to indicate that early-onset CRC cases are highly associated with mutation signatures that are caused by exposure to Colibactin, a mutagen produced by certain strains of *Escherichia coli* [32]. These findings came from a large study evaluating the CRC genomes of 981 patient samples from eleven countries. The mouse studies evaluated here did not report elevated levels of colibactin producing *Escherichia coli* (*E. coli*), however this may represent a fundamental difference in microbiomes between species [15, 18, 21]. Further work is needed to evaluate what role MNPLs may have in promoting the growth of colibactin producing *E. coli* in humans.

### MNPLs induce known tumorigenesis mechanisms in cell and animal models

The findings from the studies using mouse models were striking, in that all of them reported that exposure to MNPLs was associated with increased tumor load compared to controls [14, 18, 19, 23]. Yang and colleagues (2023) even found that MNPLs had an influence over suppressing the immune system in their CT26-Luc carcinoma injected mice, demonstrating that the impact of MNPLs in CRC development is complex and is not restricted to the colonocyte [23]. While the findings from mouse studies are clinically important, it must be stressed that these experiments were conducted using models of colitis-associated carcinoma (AOM + DSS), or in wild type mice injected with colon cancer cells. Thus, both mouse models are expected to develop colon cancer, meaning that tumor loads in MNPL exposed mice were compared relative to a baseline amount of colon tumors [33]. Healthy wildtype mice fed MNPLs do not go on to develop colorectal tumors or amass any DNA damage [15, 19], however they do have shortening of their gastrointestinal tracts relative to mice not exposed to MNPLs [15]. Other studies report similar findings, whereby even though transcriptional and molecular changes were observed in healthy colons exposed to MNPLs, it failed to translate to overt disease and development of CRC [34, 35]. This suggests that MNPLs influence over tumorigenesis relies on an already compromised gastrointestinal barrier, which is in keeping with the literature stating that CRC is most likely to be induced by MNPLs in instances of chronic colon inflammation [1]. Individuals with inflammatory bowel disease, or compromised gut barriers from other causes (smoking, alcohol, stress, infections, poor diets, and medications) are perhaps the most at risk to the effects of MNPLs and should perhaps take measures to reduce MNPL consumption [36].

Dysbiosis was reported after MNPLs exposure in the two studies using the colitis-associated carcinoma mouse models (AOM + DSS) [18, 19], and in one study on healthy wildtype mice [15]. Pan, Han, and colleagues (2025) report increases in *Lachnoclostridium* and *Clostridiales* species [18], while Tian and colleagues (2025) observed increases in *Allobaculum*, but decreases in *Lactobacillus* [19]. The differences observed between the two studies could be a result of genetic or environmental differences in the mice used, or may even be a direct influence of the type and size of the MNPLs used – however, MNPL type was not reported by Pan, Han, and colleagues (2025) [18]. The wildtype mice fed MNPLs had increased *Desulfovibrio*, which was positively correlated with biotin production and sulphur-relay pathways [15].

Elevated levels of *Lachnoclostridium* and *Allobaculum* are known to be associated with CRC in humans and mice, respectively [37, 38], while *Lactobacillus* and *Clostridiales* have been shown to have CRC fighting properties [39, 40]. Generally, these changes to the microbiome favour the development of CRC, apart from the increase in *Clostridiales*. Although the alterations to the microbiome are interesting and may reflect a potential mechanism for CRC tumorigenesis [12], it should be noted that changes to the microbiome do not themselves demonstrate a causal relationship between altered microbiome and cancer development. In fact, the addition of MNPLs to this equation further blurs the link between these variables, meaning that further research is required to delineate the relationship between MNPLs, the microbiome, and CRC.

The most prevalent finding in animal and cell models was the production of reactive oxygen species (ROS). It was reported in five studies using different MNPLs on colonocyte cell lines (Caco-2 and HT29), as well as in one study that assessed a macrophage cell line (RAW264.7). ROS plays a dual role in CRC, where initially higher than normal levels of ROS favour the development of colorectal cancer by causing cellular damage [41], DNA damage, and inflammation. There is however an upper limit beyond which ROS overproduction can cause cell death making this a potential target for cancer therapeutics [42]. Increased protective autophagy, and inhibition of pyroptosis were identified as mechanisms of evading cell death after MNPL exposure, which may help to explain initial neoplastic proliferation in addition to their observed resistance to oxaliplatin chemotherapy [14, 18]. Only two studies went on to further evaluate DNA integrity. Exposure to 20 nm PS nanoplastics was found to increase ROS and induce DNA damage [19]. Notably, they were further able to validate these *in vitro* findings in their AOM + DSS colitis mouse model. The other study to assess this relationship utilized only the chemical leachates from environmentally weathered plastics (i.e. no direct MNPL application to cell cultures). These results were variable in that only nylon leachate increased ROS and induced DNA damage, whereas polyvinyl chloride (PVC) and high-density polyethylene (HDPE) induced DNA damage without increasing ROS [20]. This may suggest another mechanism by which the chemicals bound to MNPLs can interfere with DNA integrity that is independent of ROS production.

It’s also important to note that MNPL exposure in HCT116, SW480, and LoVo colorectal cancer cell lines resulted in increased cell proliferation and invasion [14, 18]. Although this was observed *in vitro*, this may translate to a more aggressive cancer type *in vivo* with a higher risk of rapid growth, ability to invade local tissue and eventually metastasize. This provides further support for the recent evidence that MNPLs can promote metastasis of CRC to liver [43].

When we compare the MNPL types used in cell and mouse experiments with the MNPL types found in humans, only PE (and to a lesser extent, PS) is common amongst them. One study reports PE as the second most abundant MNPL in CRC tissue [18] (while the other two do not report relative abundance [13, 21]) and another finds that PE is the third most abundant type in feces of both CRC patients and healthy controls. Conversely PS is noted to be the 6th most abundant MNPL in feces in both groups, though is proportionately much lower in CRC samples (< 1% compared to 5.7% in healthy controls). Based on what we have gathered from cell lines and mouse experiments, it’s possible that PE plays a role in influencing CRC pathogenesis via promoting macrophage ROS production [23], and increasing colonocyte lactate dehydrogenase and superoxide dismutase production [24]. PS may also play a role, as it has been well shown to promote activation of known carcinogenic mechanisms in cells and mice (ROS production and DNA damage, etc. [15, 19]), however its presence in CRC tissue and feces is less well characterized. The common use of PS in laboratory experiments may instead be more reflective of it being cheap and easy to acquire from commercial sources. All other MNPLs types predominantly found in humans have yet to be explored mechanistically in an experimental setting, and we suggest that future research aim to bridge this gap in knowledge.

### The current challenges of MNPLs research and its limitations

Collectively, the results from the studies reviewed here do provide evidence that MNPLs can induce changes in colonocytes that favour development of cancer. However, there were no instances in which any of these studies demonstrated the *de novo* development of CRC in healthy mouse colons. Instead, the only instances of CRC occurring after exposure to MNPLs in mice is when it is measured above a baseline number of tumors in models of CRC. This makes it difficult to draw conclusions about MNPLs intrinsic ability to cause cancer in mouse colons that are not already inflamed. We also remark that there have been other reports that conflict with the *in vitro* findings, whereby exposure to MNPLs did not affect ROS or DNA integrity [44]. The variability of results in MNPLs research stems from the fact that many different types of plastics and particle sizes are employed in the methodologies. This highlights the need for stricter reporting when it comes to conducting research using MNPLs. Guidelines for how to conduct research on MNPLs should be made available, with a template for researchers to complete regarding the source, size, type, and purity of MNPLs, as well as any sources of contamination encountered throughout the experiment (e.g. from plastic labware or instruments). This type of information is essential to standardizing research using MNPLs, which can help to reduce costs and redundancy in research.

The data pertaining to MNPLs and CRC in humans is only correlational at this point, and reflects the findings from small samples sizes, mostly from China. We know that MNPLs are absorbed by the body and get deposited in organs via the circulatory system. However, we still do not know if/how MNPLs influence development of disease [18, 45]. Furthermore, investigating the causal relationship of MNPLs on disease development in humans is difficult, as it is not ethical to conduct experimental studies on human subjects considering the preliminary knowledge on MNPLs indicates they have toxic effects. To more thoroughly investigate this relationship going forward, much larger longitudinal and epidemiological studies involving humans are necessary.

## Conclusion

At this stage, there is insufficient research evaluating MNPLs in early-onset CRC, so it remains uncertain whether MNPLs specifically play a role in the higher incidence of early-onset CRC cases. However, the evidence of MNPL’s role in the development of CRC in general continues to emerge, and it seems likely that the population most at risk for MNPL-induced CRC are those with chronically disrupted gastrointestinal barriers.

The key mechanisms associated with MNPL-CRC tumorigenesis are the increased production of ROS, DNA damage, as well as a potential role of dysbiosis. Human studies describe higher amount of MNPLs within CRC tumors and in the feces from CRC patients. While we are still uncovering the health effects of MNPLs, the preliminary evidence largely points to MNPLs exerting toxic effects on the body. Although we are still in the early days of research, it may be wise to implement mandates around reducing plastic use in food and beverage packaging, and to label the types of plastics used for food and beverage packaging. As our understanding of MNPLs influence on health continues to grow, we should prepare to take action to reduce our MNPL load by increasing public awareness and advocating for policy change to eliminate plastic pollution and reduce plastic production.

## Data Availability

No datasets were generated or analysed during the current study.

## Appendix

**Table 2:** Search on Ovid MEDLINE ALL: October 26th, 2025

**Colorectal neoplasm**	**Microplastic**
Colorectal cancer*	mesoplastic*
Colorectal carcinoma*	microparticle* AND plastic*
cancer*, colorectal	microplastic*
carcinoma*, colorectal	nanoparticle* AND plastic*
colorectal neoplasm*	plastic microparticle*
colorectal tumo?r*	plastic nanoparticle*
neoplasm*, colorectal	
tumo?r*, colorectal	

Boolean operator used: “Column A” AND “Column B”

**PubMed search strategy on October 26th, 2025:** (Colorectal neoplasm[MeSH Terms]) AND (microplastic[MeSH Terms])

## Abbreviations

*ABS*: Acrylonitrile butadiene styrene
*AOS + DSS*: Azoxymethane and dextran sulphate sodium
*CRC*: Colorectal cancer
*HDPE*: High density polyethylene
*IBD*: Inflammatory bowel disease
*LDH*: Lactate dehydrogenase
*MP*: Microplastic
*MNPLs*: Micro- and nano-plastics
*NP*: Nanoplastic
*PA*: Polyamide
*PAH*: Polycyclic aromatic hydrocarbons
*PE*: Polyethylene
*PET*: Polyethylene terephthalate
*PFOS*: Perfluorooctane sulfonate
*PP*: Polypropylene
*PS*: Polystyrene
*PU*: Polyurethane
*PVC*: Polyvinyl chloride
*ROS*: Reactive oxygen species
